# Mutated *FANCA* Gene Role in the Modulation of Energy Metabolism and Mitochondrial Dynamics in Head and Neck Squamous Cell Carcinoma

**DOI:** 10.3390/cells11152353

**Published:** 2022-07-30

**Authors:** Nadia Bertola, Paolo Degan, Enrico Cappelli, Silvia Ravera

**Affiliations:** 1 Department of Experimental Medicine, University of Genoa, Via De Toni 14, 16132 Genova, Italy; nadia.bertola@edu.unige.it; 2 U.O. Mutagenesi IRCCS Policlinico San Martino—IST (Istituto Nazionale per la Ricerca sul Cancro), Largo Rosanna Benzi 10, 16132 Genova, Italy; paolo.degan@hsanmartino.it; 3 Haematology Unit, IRCCS Istituto Giannina Gaslini, Via Gerolamo Gaslini 5, 16148 Genova, Italy; enricocappelli@gaslini.org

**Keywords:** anaerobic glycolysis, antioxidant defences, autophagy, double-strand DNA damage, Fanconi Anaemia, HNSCC, mitochondrial fusion and fission, mitophagy, oxidative phosphorylation, oxidative stress

## Abstract

Fanconi Anaemia (FA) is a rare recessive genetic disorder characterized by a defective DNA repair mechanism. Although aplastic anaemia is the principal clinical sign in FA, patients develop a head and neck squamous cell carcinoma (HNSCC) with a frequency 500–700 folds higher than the general population, which appears more aggressive, with survival of under two years. Since FA gene mutations are also associated with a defect in the aerobic metabolism and an increased oxidative stress accumulation, this work aims to evaluate the effect of *FANCA* mutation on the energy metabolism and the relative mitochondrial quality control pathways in an HNSCC cellular model. Energy metabolism and cellular antioxidant capacities were evaluated by oximetric, luminometric, and spectrophotometric assays. The dynamics of the mitochondrial network, the quality of mitophagy and autophagy, and DNA double-strand damage were analysed by Western blot analysis. Data show that the HNSCC cellular model carrying the *FANCA* gene mutation displays an altered electron transport between respiratory Complexes I and III that does not depend on the OxPhos protein expression. Moreover, *FANCA* HNSCC cells show an imbalance between fusion and fission processes and alterations in autophagy and mitophagy pathways. Together, all these alterations associated with the *FANCA* gene mutation cause cellular energy depletion and a metabolic switch to glycolysis, exacerbating the Warburg effect in HNSCC cells and increasing the growth rate. In addition, the altered DNA repair due to the *FANCA* mutation causes a higher accumulation of DNA damage in the HNSCC cellular model. In conclusion, changes in energy metabolism and mitochondrial dynamics could explain the strict correlation between HNSCC and FA genes, helping to identify new therapeutic targets.

## 1. Introduction

Fanconi Anaemia (FA) is a rare recessive autosomal or X-linked genetic disorder [1]. So far, 23 genes are associated with FA, although mutations within *FANCA*, *FANCC*, or *FANCG* genes are the most frequent [2,3]. Progressive bone marrow (BM) failure and aplastic anaemia, with a 5000-fold increased risk [4], are the leading causes of death in FA patients due to the defective DNA repair system [5]. Moreover, the FA phenotype is characterized by various congenital malformations, metabolic dysfunctions susceptibility, and increased risk of malignancies such as leukaemia and squamous cell carcinoma [2], including the head and neck squamous cell carcinoma (HNSCC), the most common solid tumour in FA patients [6]. HNSCC is the sixth most common cancer worldwide [7] and is the most common tumour in the head and neck, developing from the pharynx, larynx, and oral cavity mucosal epithelium in the general population [8]. HNSCC onset depends on several risk factors, including tobacco smoking, alcohol consumption, and HPV infection [9]. Nevertheless, FA patients can develop HNSCC even without exposure to the most common risk factors for this type of cancer and have a 500–700-fold higher risk of developing an HNSCC than the general population [10]. HNSCC in FA patients is more aggressive, with survival under two years, and arises at a significantly younger age [11,12,13]. On the other hand, FA genetic mutations cause defects in DNA repair systems, and genomic and chromosomal instability [11,12,13], contributing to cancer progression [14]. Higher percentages of altered *FANC* genes have been found in HNSCC patients unaffected by FA [15,16,17,18], as well as in various cancer types, both sporadic and hereditary: among them, breast and ovarian cancer with mutations occurring most likely in *FANCA*, *FANCS/BRCA1*, and *FANCD2/BRCA2* genes [19,20].

Over the last decade, in addition to a defective DNA repair system, morphological and functional mitochondrial defects have been associated with FA pathogenesis [21]. Specifically, mutated FA genes are associated with altered mitochondrial biogenesis and dynamics and metabolic dysfunctions, which cause lipids accumulation and unbalanced oxidative stress [22,23,24]. In FA cells, a defective electron transport between complexes I and III causes dysfunction in oxidative phosphorylation (OxPhos) [24,25] that induces a metabolic shift from aerobic to anaerobic metabolism and a decrease in cellular energy status [24,25]. 

Although FA mitochondrial defects have been characterized mainly in lymphoblasts and fibroblasts, mitochondria morphologic and metabolic characterization is missing in FA-associated HNSCC. Thus, we aim to provide data to fill this gap, investigating how FA-related mitochondrial dysfunctions influence the HNSCC energy metabolism and mitochondrial dynamics. For this scope, an HNSCC cell line carrying *FANCA* gene mutation (OHSU-974-S91) and an HNSCC cell line with the functional *FANCA* gene inserted (OHSU-974-FAcorr) were employed. Spectrophotometric, oximetric, luminometric, and Western blot assays were performed to evaluate oxidative phosphorylation; respiratory complexes expression and functionality; cellular energy state; mitochondrial biogenesis and dynamics, autophagy, and mitophagy processes; oxidative stress damages; antioxidant defences; and DNA damages accumulation.

## 2. Materials and Methods

### 2.1. Cell Lines and Culture Conditions 

OHSU-974-S91 (OHSU-S91) and OHSU-974-FAcorr (OHSU-FAcorr) are human head and neck squamous cell carcinoma (HNSCC) cell lines kindly provided by prof. Susanne Wells, Cincinnati Children’s Hospital Medical Center Cincinnati, USA. Specifically, OHSU-S91 carries *FANCA* gene mutation, while OHSU-FAcorr is an isogenic HNSCC cell line corrected with the functional *FANCA* gene inserted with a retrovirus. Cells were grown in RPMI medium supplemented with 10% foetal calf serum and antibiotics (100 U/mL penicillin and 100 µg/mL streptomycin) at 37 °C with a 5% CO_2_ [26].

### 2.2. Oxygen Consumption Assay 

Oxygen consumption was measured with an amperometric electrode (Unisense Microrespiration, Unisense A/S, Denmark) in a closed chamber at 25 °C. For each experiment, 2 × 10^5^ cells were resuspended in phosphate buffer saline (PBS) and permeabilized with 0.03 mg/mL digitonin for 1 min. To stimulate the pathways composed of complexes I, III, and IV or II, III, and IV, 10 mM pyruvate plus 5 mM malate or 20 mM succinate were employed, respectively [18,24]. To test the cellular affinity for glucose, glutamine, and fatty acids as respiratory substrates, 3 µM BPTES (a glutaminase inhibitor [27]), 4 µM Etomoxir (a fatty acid oxidation inhibitor [28]), and 2 µM UK5099 (a mitochondrial pyruvate carrier inhibitor [29]) were added to cells resuspended in the growth medium. Data were expressed as nmol O/min/10^6^ cells.

### 2.3. F_o_F_1_ ATP-Synthase Activity Assay 

The F_o_F_1_ ATP-synthase (ATP Synthase) activity was evaluated incubating 2 × 10^5^ cells at 25 °C for 10 min in a medium containing: 50 mM Tris-HCl (pH 7.4), 50 mM KCl, 1 mM EGTA, 2 mM MgCl_2_, 0.6 mM ouabain, 0.25 mM di(adenosine)-5-Penta-phosphate (an adenylate kinase inhibitor), and 25 μg/mL ampicillin (0.1 mL final volume); then, 10 mM pyruvate plus 5 mM malate or 20 mM succinate were employed to stimulate complexes I, III, and IV or complexes II, III, and IV pathways, respectively [17]. As for OCR evaluation, 3 µM BPTES, 4 µM Etomoxir, and 2 µM UK5099 were used for the cellular energy substrate affinity evaluation. In this case, cells were suspended in the growth medium diluted 1:1 with the solution described above. In each case, ATP synthesis was induced by adding 0.1 mM ADP. The reaction was monitored every 30 s for 2 min with a luminometer (GloMax^®^ 20/20 Luminometer, Promega Italia, Milano, Italy), using the luciferin/luciferase chemiluminescent method (luciferin/luciferase ATP bioluminescence assay kit CLS II, Roche, Basel, Switzerland). ATP standard solutions in a concentration range between 10^−8^ and 10^−5^ M were used for calibration. Data were expressed as nmol ATP/min/10^6^ cells [24]. 

### 2.4. P/O Ratio 

P/O value is the ratio of aerobic ATP synthesis and oxygen consumption and represents a parameter of OxPhos efficiency. Efficient mitochondria have a P/O value of around 2.5 or 1.5, activating the pathways leading by complexes I or II, respectively. Conversely, a lower P/O ratio suggests that part of the oxygen is not employed for energy production but may contribute to the formation of reactive oxygen species (ROS) [30]. 

### 2.5. Cell Homogenate Preparation 

Cells were centrifuged at 1000 rpm for 5 min, and the growth medium was removed. The pellet was washed in PBS twice and centrifuged again. Pellet was resuspended in Milli-Q water plus protease inhibitor and sonicated in ice twice for 10-s, with a 30-s break to prevent the mixture from warming, using the Microson XL Model DU-2000 (Misonix Inc., Farmingdale, NY, USA). Total protein content was estimated with the Bradford method [31]. 

### 2.6. Lactate Dehydrogenase Activity Assay 

Lactate dehydrogenase (LDH) activity was assayed spectrophotometrically following NADH oxidation at 340 nm. The assay mix contained Tris-HCl (pH 7.4), 1 mM pyruvate, and 0.2 mM NADH [24]. 

### 2.7. Glucose Consumption and Lactate Release Assay 

Glucose consumption was evaluated in the growth medium, following NADP reduction at 340 nm. Then, 10 µL of growth medium was added to 50 mM Tris-HCl pH 8.0, 1 mM NADP, 10 mM MgCl_2_, and 2 mM ATP. Samples were analysed spectrophotometrically before and after the addition of 4 μg of purified hexokinase plus glucose-6- phosphate dehydrogenase [32]. 

Lactate concentration in the growth medium was assayed spectrophotometrically, following the reduction of NAD^+^, at 340 nm. The assay medium contained: 10 µL of the growth medium, 100 mM Tris- HCl pH 8, 5 mM NAD^+^, and 1 IU/mL of lactate dehydrogenase. Samples were analysed spectrophotometrically before and after the addition of 4 μg of purified lactate dehydrogenase [32]. Both data were normalized on the cell number. 

The glycolysis rate was calculated as the percentage of real released lactate on the theoretical lactate production, which corresponds to twice the concentration of glucose consumed (as in an exclusive anaerobic metabolism, one glucose molecule is converted into two lactate molecules). 

### 2.8. Respiratory Complexes Enzymatic Activities 

Complex I and Complex II activities were assayed spectrophotometrically at 420 nm following the ferricyanide reduction. The reaction mix contained: 100 mM Tris-HCl (pH 7.4), 0.8 mM K_3_[Fe(CN)_6_], and 0.7 mM NADH for Complex I or 20 mM succinate for Complex II. 

Complex III activity was assayed following the oxidized Cytochrome c (Cyt c) reduction at 550 nm. The reaction mix contained: 100 mM Tris-HCl (pH 7.4), 0.5 mM NADH, and 0.03% oxidized cytochrome c. 

Complex IV was assayed following the ascorbate-reduced Cyt c oxidation at 550 nm. The reaction mix contained: 100 mM Tris-HCl (pH 7.4), 50 µM antimycin A, and 0.03% Cyt c reduced with 3 mM ascorbic acid [33].

### 2.9. ATP and AMP Intracellular Content Evaluation and ATP/AMP Ratio Calculation 

For each assay, 50 μg of total protein was used. ATP was assayed spectrophotometrically following NADP reduction at 340 nm. Assay medium contained: 100 mM Tris-HCl (pH 8.0), 0.2 mM NADP, 5 mM MgCl_2_, and 50 mM glucose. Samples were analysed before and after the addition of 3 μg of purified hexokinase plus glucose-6-phosphate dehydrogenase. AMP was assayed spectrophotometrically following NADH oxidation at 340 nm. Reaction medium contained: 100 mM Tris-HCl (pH 8.0), 5 mM MgCl_2_, 0.2 mM ATP, 10 mM phosphoenolpyruvate, 0.15 mM NADH, 10 IU adenylate kinase, 25 IU pyruvate kinase, and 15 IU of lactate dehydrogenase. ATP/AMP value was calculated as the ratio between the intracellular concentration of ATP and AMP, expressed in mM/mg of total protein [18,24]. 

### 2.10. Western Blot Analysis 

For Western blot analysis, 30 μg of proteins were loaded for each sample to perform denaturing electrophoresis (SDS-PAGE) on 4–20% gradient gels (Bio-Rad, Hercules, CA, USA). The following primary antibodies were used: anti-ND1 (Abcam, Cambridge, UK #ab181848), anti-SDHB (Abcam, Cambridge, UK #ab84622), anti-MTCO2 (Abcam, Cambridge, UK #ab79393), anti-ATP synthase β subunit (Sigma-Aldrich, St. Louis, MI, USA #HPA001520), anti-CLUH (Bethyl Lab. Inc., Waltham, MA, USA #A301-764A), anti-DRP1 (ThermoFisher, Waltham, MA, USA #DRP1-101AP), anti-MFN2 (ThermoFisher, Waltham, MA, USA #PA5-72811), anti-OPA1 (Sigma-Aldrich, St. Louis, USA #HPA036926), anti-Beclin1 (Cell Signaling, Danvers, MA, USA #3495P), anti-Atg7 (Cell Signaling, Danvers, MA, USA (D12B11) #8558P), anti-Atg12 (Cell Signaling, Danvers, MA, USA (D88H11) #4180P), anti-Atg16L1 (Cell Signaling, Danvers, MA, USA (D6D5) #8089P), anti-LC3 (Novus Biologicals, Minneapolis, MI, USA #NB100-2220), anti-Pink1 (ThermoFisher, Waltham, MA, USA #PA1-4515), anti-Parkin (ThermoFisher, Waltham, MA, USA #PA5-13399), anti-G6PD (Abcam, Cambridge, UK #ab124738), anti-H6PD (Abcam, Cambridge, UK #ab170895), anti-phosphorylated-γ-H2AX (Merk-Millipore, Burlington, VT, USA #05-636), and anti-Actin (Santa Cruz Biotechnology, Dallas, TX, USA #sc-1616). All primary antibodies were diluted 1:1000 in PBS plus 0.15% tween (PBSt). Specific secondary antibodies were employed (Sigma-Aldrich, St. Louis, MI, USA), all diluted 1:10,000 in PBSt. Bands were detected and analysed for optical density using an enhanced chemiluminescence substrate (ECL, Bio-Rad, Hercules, CA, USA), a chemiluminescence system (Alliance 6.7 WL 20M, UVITEC, Cambridge, UK), and UV1D software (UVITEC, Cambridge, UK). All the bands of interest were normalized with Actin levels detected on the same membrane. 

### 2.11. Malondialdehyde Evaluation 

Malondialdehyde (MDA) concentration was assessed to evaluate lipid peroxidation, using the thiobarbituric acid reactive substances (TBARS) assay. This test is based on the reaction of thiobarbituric acid (TBA) with MDA, a breakdown product of lipid peroxides. The TBARS solution contained 26 mM thiobarbituric acid and 15% trichloroacetic acid (TCA) in 0.25 N HCl. To evaluate MDA concentration, 50 μg of total protein dissolved in 300 μL of Milli-Q water was added with 600 μL of TBARS solution. The mix was incubated at 95 °C for 60 min. The sample was then centrifuged at 14,000 rpm for 2 min, and the supernatant was then analysed spectrophotometrically at 532 nm [18,24]. 

### 2.12. Enzymatic Antioxidant Defences Assay 

Glutathione reductase (GR) activity was assayed spectrophotometrically at 340 nm, following the oxidation of NADPH. The assay medium contained: 100 mM Tris-HCl (pH 7.4), 1 mM EDTA, 5 mM GSSG, and 0.2 mM NADPH [26]. 

Catalase activity was spectrophotometrically assayed following the H_2_O_2_ decomposition at 240 nm. The assay mix contained: 50 mM phosphate buffer (pH 7.0) and 5 mM H_2_O_2_. 

In total, 20 μg of total protein was used for both assays. Data were normalized on the sample protein content [24]. 

Glucose 6-phosphate dehydrogenase (G6PD) and hexose 6-phosphate dehydrogenase (H6PD) activity were assayed spectrophotometrically at 340 nm following NADP reduction. The assay mix contained Tris-HCl (pH 7.4), 0.5 mM NADP, and 10 mM glucose-6-phosphate or glucose for G6PD and H6PD dosage, respectively [34]. 

### 2.13. DNA Damages Induction 

Both OHSU-974-FAcorr and OHSU-974-S91 were seeded in 6 Multi-Well Plates and cultured till confluence, as described in Section 2.1. Furthermore, cells were treated for 3 h with 2 mM hydroxyurea (HU) dissolved in a fresh medium to induce DNA damage. Control cells seeded in parallel received only fresh medium. Cells were then collected and prepared for WB analysis as described in Section 2.12 [35].

### 2.14. Statistical Analysis

Data were analysed appropriately using unpaired *t*-test or two-way ANOVA, using Prism 8 Software. Data are expressed as mean ± standard deviation (SD) and are representative of at least three independent experiments. An error with a probability of *p* < 0.05 was considered significant.

## 3. Results

### 3.1. FANCA Gene Mutation Negatively Affects the Aerobic Metabolism, Increasing the Uncoupling between the Oxygen Consumption Rate and the ATP Synthesis, and Lactate Fermentation, Causing a Depletion in the Energy Status

**Figure 1:**
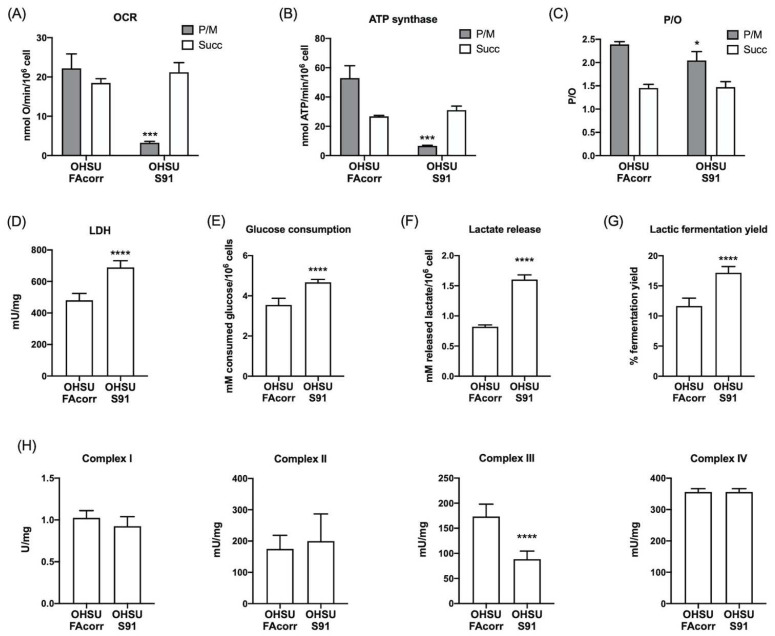
Aerobic metabolism and lactic fermentation in OHSU-974-FAcorr and OHSU-974-S91 cells. (**A**) Oxygen consumption rate (OCR). (**B**) Aerobic ATP synthesis through F_o_F_1_ ATP-synthase. For both graphs, data were obtained using pyruvate plus malate (grey columns) or succinate (white columns) as respiring substrates. (**C**) P/O value, an indicator of OxPhos efficiency, is calculated as the ratio between the synthesized ATP and the OCR. (**D**) Lactate dehydrogenase (LDH) activity. (**E**) Glucose consumption. (**F**) Lactate release in the growth medium. (**G**) Lactic fermentation yield. (**H**) Mitochondrial respiratory complexes activity. Data are reported as mean ± SD, and each graph is representative of at least 3 independent experiments. Statistical significance was tested opportunely with the unpaired *t*-test or two-way ANOVA. *, ***, and **** represent a *p* < 0.05, 0.001, and 0.0001, respectively, between OHSU-974-S91 cells and the OHSU-974-FAcorr cells, used as control.

To evaluate mitochondrial energy metabolism, oxygen consumption rate (OCR) and ATP synthesis were analysed in OHSU-FAcorr and OHSU-S91 cell lines. The results, reported in Figure 1, show that, in OHSU-FAcorr, both respiratory complex pathways sustained OCR (Figure 1A) and ATP synthesis (Figure 1B), with a prevalence of complex I-triggered pathway. In contrast, in OHSU-S91, OCR and ATP synthesis stimulated by pyruvate plus malate appear negligible, and OxPhos is principally sustained by succinate, confirming the altered electron transfer between complexes I and III, typical of mutated *FANCA* cells [25]. In addition, OHSU-FAcorr OxPhos appears completely coupled as P/O values are around 2.5 and 1.5, after stimulation with pyruvate plus malate or succinate, respectively. Conversely, in OHSU-S91, a slight decrement in the P/O value associated with the complex I pathway is observed (Figure 1C), suggesting a partial uncoupling between OCR and ATP synthesis. 

Usually, cancer cells display high anaerobic glucose catabolism, namely the Warburg effect, associated with a faster cellular proliferation [36]. Thus, lactate dehydrogenase (LDH) activity was assayed to evaluate whether lactic fermentation has been increased in *FANCA* mutated HNSCC cells to compensate for mitochondrial dysfunction. Data (Figure 1D) show that, in OHSU-S91, LDH activity is significantly higher than in OHSU-FAcorr, suggesting an enhancement of the metabolic switch to the anaerobic metabolism. The increase in anaerobic glucose catabolism is confirmed by the evaluation of glucose consumption, lactate release, and subsequent lactic fermentation yield, shown in Figure 1E–G. Specifically, OHSU-S91 cells show a higher glucose consumption and lactate release compared to OHSU-FAcorr, resulting in an approximately 50% increase in lactic fermentation in OHSU-S91.

**Figure 2:**
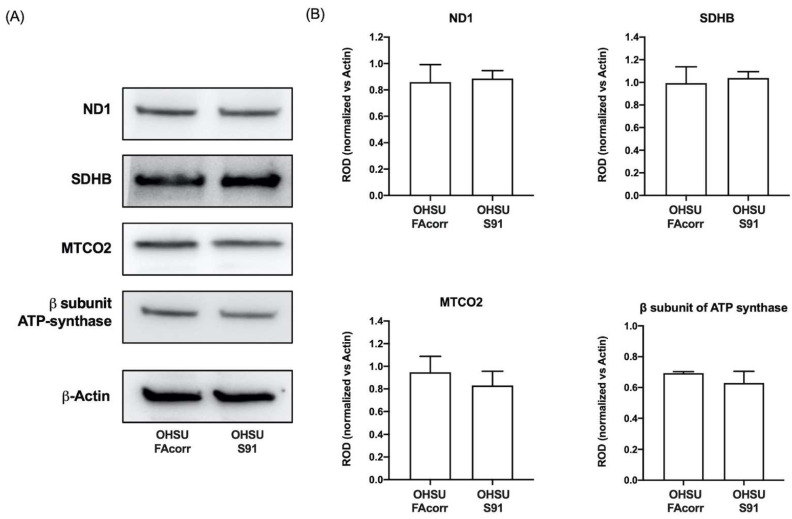
Expression of OxPhos proteins in OHSU-974-FAcorr and OHSU-974-S91 cells. (**A**) Western blot (WB) signals of OxPhos subunits (e.g., namely ND1 (Complex I), SDHB (Complex II), MTCO2 (Complex IV) and β-subunit of ATP synthase), and β-Actin, used as the housekeeping protein. Each signal is representative of at least 3 independent experiments. (**B**) Densitometric analysis of WB signals reported in Panel A normalized versus the housekeeping signal. Data are reported as mean ± SD of Relative Optical Density (ROD), and each graph is representative of at least 3 independent experiments. Statistical significance was tested opportunely with the unpaired *t*-test, and no significant differences are observed between OHSU-974-S91 cells and the OHSU-974-FAcorr cells.

To understand if the dysfunctional aerobic metabolism depends on a defect in respiratory complexes’ function, their activities were measured spectrophotometrically in both cell lines (Figure 1H). No significant differences in complexes I, II, and IV activity were observed comparing the two cell lines. By contrast, the data show a statistically significant decrease in complex III activity in OHSU-S91 compared to OHSU-FAcorr when evaluated by NADH addition but not in the presence of succinate (Figure 1H and Figure S1). Since the respiratory complex III activity is measured by assaying the electron transport from upstream complexes, the impairment observed in the presence of NADH, but not of succinate, confirms that the weak point is the electron transfer between the first and third complexes and not complex III itself, as already observed in FA lymphoblasts [25]. Nevertheless, no differences are observed in the protein expression of ND1 (Complex I), SDHB (Complex II), MTCO2 (Complex IV), and ATP synthase β subunit between OHSU-S91 and OHSU-FAcorr, indicating that the altered metabolism is not related to a different expression of OxPhos proteins (Figure 2A,B). 

**Figure 3:**
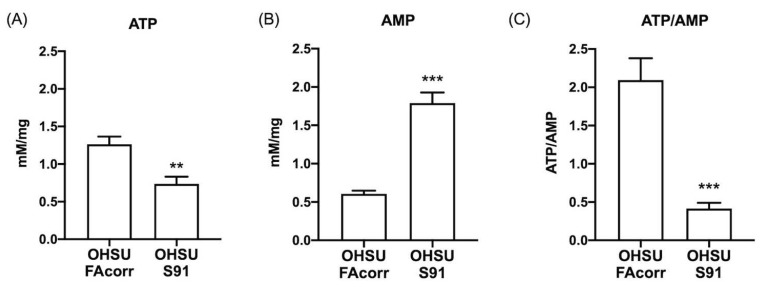
ATP and AMP intracellular concentrations and energy status in OHSU-974-FAcorr and OHSU-974-S91. (**A**) Intracellular ATP content. (**B**) Intracellular AMP content. (**C**) ATP/AMP ratio, as a marker of cellular energy status. Data are reported as mean ± SD, and each graph is representative of at least 3 independent experiments. Statistical significance was tested with an unpaired *t*-test. ** and *** represent a *p* < 0.01 and 0.001, respectively, between OHSU-974-S91 cells and the OHSU-974-FAcorr cells used as control.

Despite the lactate fermentation increment, the OxPhos impairment causes a depletion in the cellular energy status. Specifically, ATP content is halved (Figure 3A) whereas AMP concentration increases by about three-fold in OHSU-S91 compared to OHSU-FAcorr (Figure 3B), determining a four-fold reduction of the ATP/AMP ratio in OHSU-S91 compared to control cells (Figure 3C).

### 3.2. The Mutated FANCA Gene Causes a Change in the Energy Substrates Affinity

**Figure 4:**
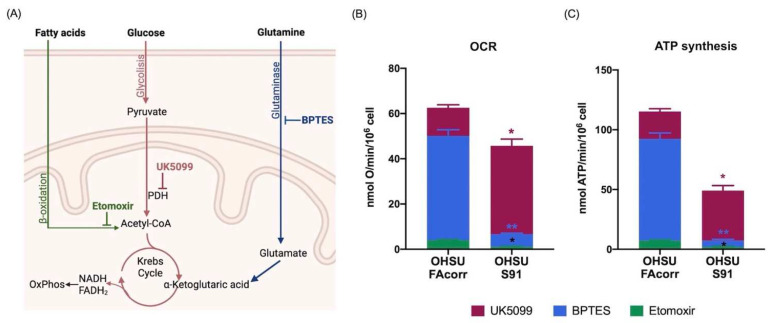
Respiratory substrates affinity in OHSU-974-FAcorr and OHSU-974-S91. (**A**) Graphical representation of the inhibitors targets used to evaluate the respiratory substrates’ affinity of OxPhos metabolism. BPTES is a glutaminase inhibitor; Etomoxir prevents the transport of fatty acids to the mitochondrion, inhibiting beta-oxidation; and UK5099 is Pyruvate Dehydrogenase (PDH) inhibitor. (**B**) The extent of OCR for glucose (inhibited by UK5099, Bordeaux), glutamine (inhibited by BPTES, Blue), and fatty acids (inhibited by Etomoxir, green). (**C**) The extent of ATP synthesis through F_o_F_1_ ATP-synthase for glucose (inhibited by UK5099, Bordeaux), glutamine (inhibited by BPTES, Blue), and fatty acids (inhibited by Etomoxir, green). Data are reported as mean ± SD, and each graph is representative of at least 3 independent experiments. Statistical significance was tested with two-way ANOVA. * and ** represent a *p* < 0.05 and 0.01, respectively, between OHSU-974-S91 cells and the OHSU-974-FAcorr cells used as control.

To determine the affinity of OHSU cell lines for the energy substrates, OCR and ATP synthesis were assayed in the presence of BPTES, Etomoxir, and UK5099 to inhibit the use of glutamine, fatty acids, or pyruvate, respectively (Figure 4A). Data confirm that OHSU-S91 display a lower OxPhos activity compared to OHSU-FAcorr and show that the employment of respiratory substrate appears different in the two samples. Specifically, in OHSU-FAcorr, both OCR (Figure 4B) and ATP synthesis (Figure 4C) are principally sustained by glutamine, followed by glucose, whereas the use of fatty acids only represents a small percentage. Conversely, in OHSU-S91, most oxidative phosphorylation depends on the glucose, reducing glutamine and FA consumption as energy substrates. 

### 3.3. Mutated FANCA Alters the Balance between Mitochondrial Fusion and Fission and Impairs the Mitophagy and Autophagy Processes

**Figure 5:**
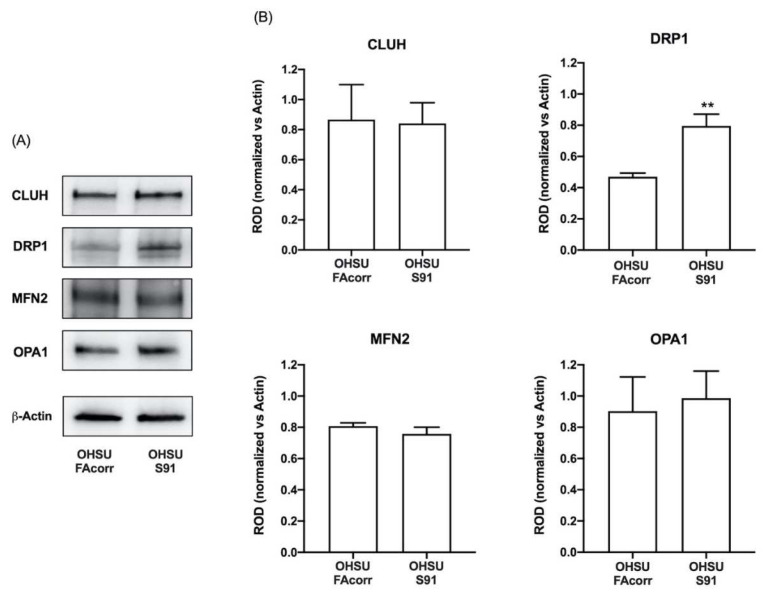
Expression evaluation of proteins involved in mitochondrial dynamics in OHSU-974-FAcorr and OHSU-974-S91 mitochondrial dynamics. (**A**) Western blot (WB) signals of CLUH (a regulator of mitochondria dynamic), DRP1 (a marker of mitochondrial fission), MFN2, OPA1 (two markers of mitochondrial fusion), and β-Actin. Each WB signal is representative of at least 3 independent experiments. (**B**) Densitometric analysis of the WB signals reported in Panel A, normalized versus β-Actin signal. Data are reported as mean ± SD, and each graph is representative of at least 3 independent experiments. Statistical significance was tested with an unpaired *t*-test. ** represents a *p* < 0.01 between OHSU-974-S91 cells and the OHSU-974-FAcorr cells used as control.

To evaluate whether the altered mitochondrial function in OHSU-S91 cells depends on defects in mitochondrial dynamics, the expression level of some proteins associated with mitochondrial fusion and fission was analysed in WB. As shown in Figure 5, the expression of CLUH, an RNA-binding protein involved in mitochondrial biogenesis [37], mitofusin 2 (MFN2), and OPA1, two mitochondrial fusion regulators [38], appear similar in OHSU-FAcorr and OHSU-S91. Conversely, the DRP1 expression, a regulatory protein involved in the mitochondrial fission [38], resulted in being almost doubled in OHSU-S91 than in OHSU-FAcorr, suggesting an imbalance in favour of the fission of the mitochondrial reticulum dynamic in HNSCCs cell expressing *FANCA* mutation. 

**Figure 6:**
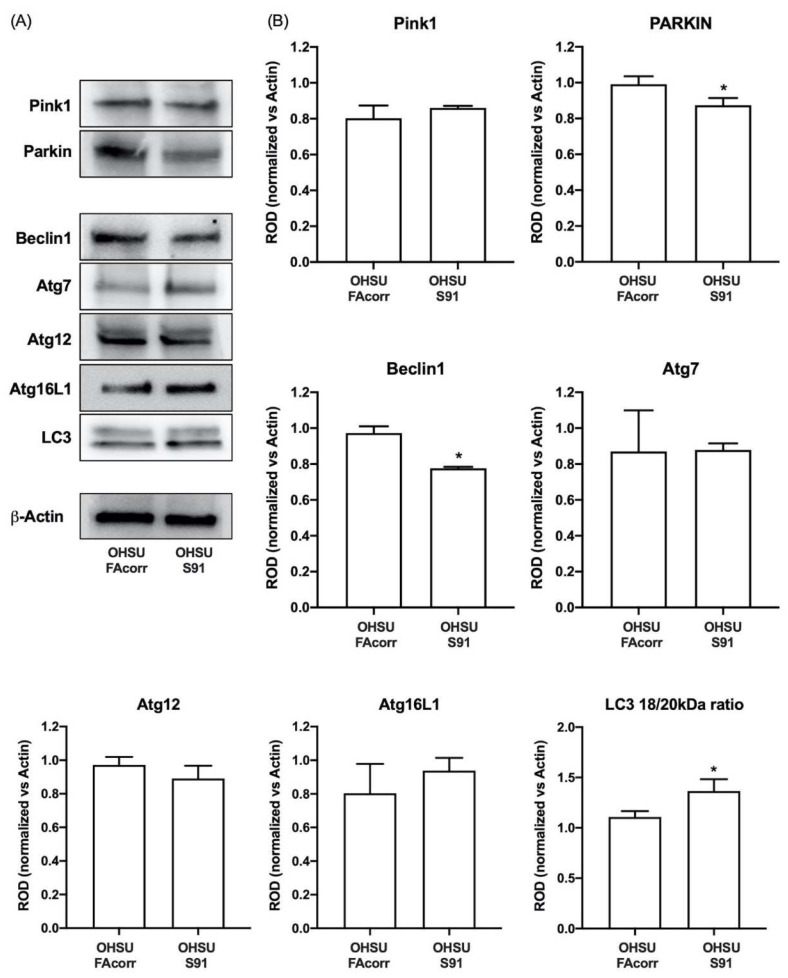
Expression evaluation of proteins involved in mitophagy and autophagy processes in OHSU-974-FAcorr and OHSU-974-S91. (**A**) Western blot signals of Pink1 and Parkin (two mitophagy markers), Beclin1, Atg7, Atg12, Atg16L1, LC3 (autophagy markers), and β-Actin. (**B**) Densitometric analysis of WB signals reported in Panel A, normalized versus β-Actin. Data in histograms are reported as mean ± SD and are representative of at least 3 independent experiments. Statistical significance was tested with an unpaired *t*-test. * represents a *p* < 0.05 between OHSU-974-S91 cells and the OHSU-974-FAcorr cells used as control.

Regarding the autophagy and mitophagy regulation, OHSU-S91 cells show LC3 activation, the common end effector of both pathways [39], as the ratio between the cleaved and un-cleaved form is 27% higher than in the control (Figure 6). However, evaluating the upstream proteins in the autophagy process, data show that Beclin1, but not its effectors, Atg7, Atg12, and Atg16L1 [40], appears less expressed in OHSU-S91 compared to OHSU-FAcorr (Figure 6). In addition, evaluating mitophagy markers expression, PINK1 level appears similar in both cell lines, but the PARKIN signal is lower in OHSU-S91 than in OHSU-FAcorr (Figure 6). Since PINK1 targets the damaged mitochondria, and PARKIN is involved in the dysfunctional mitochondria clearance via mitophagy and proteasomal mechanisms [41], these data suggest that OHSU-S91 cells can target damaged mitochondria but are not able to carry on the mitophagy process. 

### 3.4. Mutated FANCA Causes a Decrease in the Expression and Function of Enzymatic Antioxidant Defences and an Increment in Lipid Peroxidation

**Figure 7:**
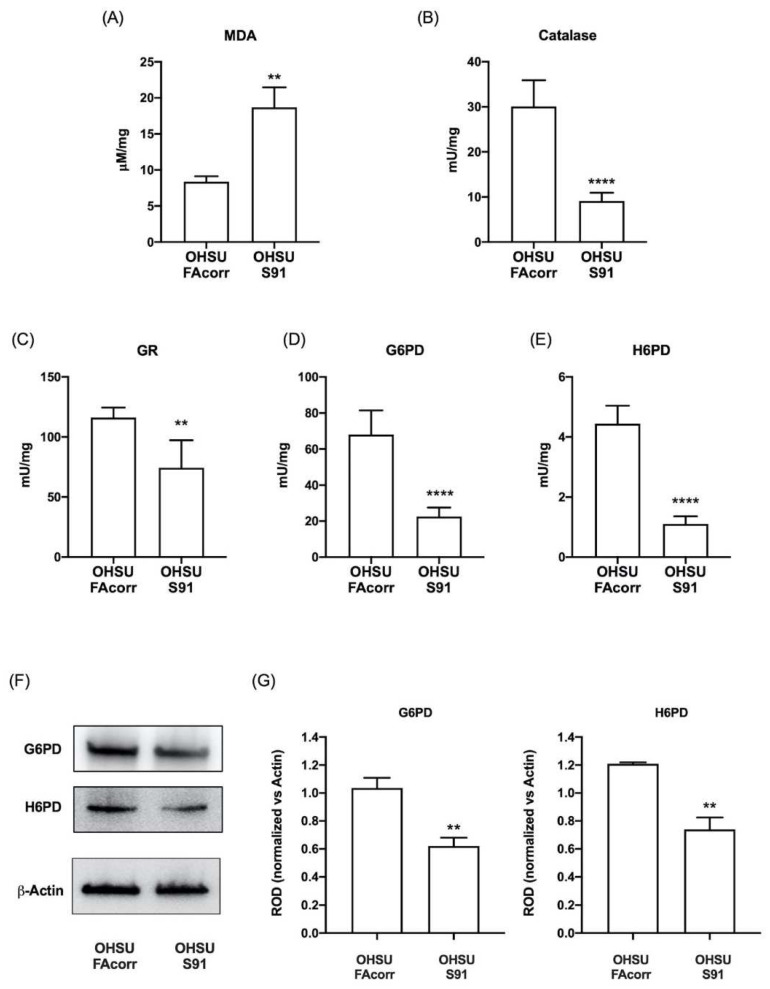
Lipid peroxidation and enzymatic antioxidant defence activities in OHSU-974-FAcorr and OHSU-974-S91. (**A**) Malondialdehyde (MDA) level, as a marker of lipid peroxidation. (**B**) Catalase activity. (**C**) Glutathione reductase (GR) activity. (**D**) Glucose-6-phosphate dehydrogenase (G6PD) activity. (**E**) Hexose-6-phosphate dehydrogenase (H6PD) activity. (**F**) G6PD and H6PD WB signals. (**G**) Densitometric analysis of WB signals reported in Panel F normalized versus β-Actin. Data are reported as mean ± SD, and each graph is representative of at least 3 independent experiments. Statistical significance was tested with an unpaired *t*-test. **, and **** represent a *p* < 0.01 and 0.0001, respectively, between OHSU-974-S91 cells and the OHSU-974-FAcorr cells used as control.

Since FA cells are characterized by elevated oxidative stress [24,42,43], to verify whether HNSCC cells carrying *FANCA* mutation display an oxidative damage increment, the malondialdehyde (MDA) level has been assayed in OHSU-FAcorr and OHSU-S91. Data in Figure 7A show that in OHSU-S91, there is a 2.5-fold increase in MDA accumulation compared to the OHSU-FAcorr. Conversely, texting the enzymatic antioxidant activities, results show a reduction of about 60% for catalase, G6PD, and H6PD activity (Figure 7B,D,E, respectively) and about 40% for GR (Figure 7C) in OHSU-S91 compared to OHSU-FAcorr. In addition, a Western blot analysis shows that the G6PD and H6PD activities reduction is associated with a decrement in their expression (Figure 7F,G).

### 3.5. Mutated FANCA Induces an Increase in Cell Proliferation but also an Additional Accumulation of Double-Strand DNA Damages

**Figure 8:**
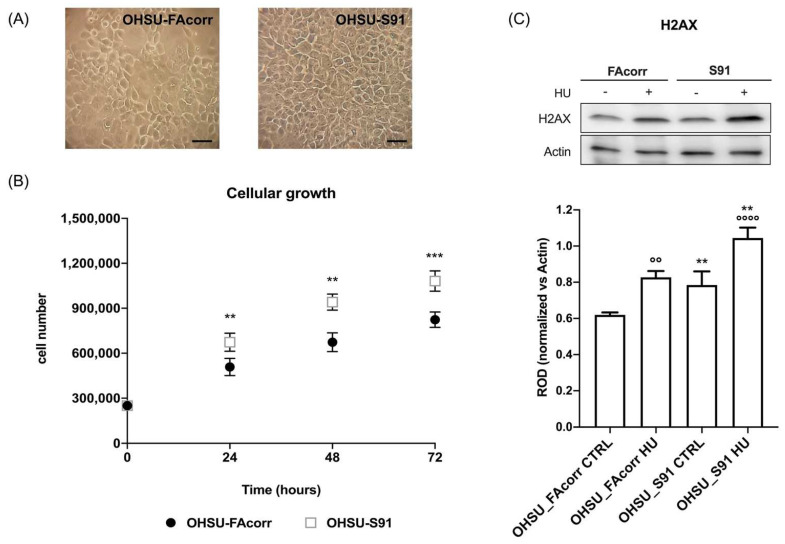
Morphology and growth curve of OHSU-974-FAcorr and OHSU-974-S91 and the evaluation of DNA double-breaks in the absence or presence of hydroxyurea. (**A**) Example of OHSU-974 FAcorr and OHSU-S91 morphology observed at the transmitted-light microscope. The scale bar corresponds to 50 μm. Each panel is representative of at least five fields of three independent experiments. (**B**) The growth curves of OHSU-974 FAcorr and OHSU-S91 were monitored for 72 h, every 24 h. (**C**) WB signal of phosphorylated-γ-H2AX (H2AX), a marker of DNA damage accumulation, in the absence or presence of hydroxyurea (HU) (upper part), and the relative densitometric analysis normalized versus β-Actin (lower part). Data are reported as mean ± SD, and each graph is representative of at least 3 independent experiments. Statistical significance was tested with a two-way ANOVA test. ** and *** represent a *p* < 0.01 and 0.001, respectively, between OHSU-974-S91 cells and the OHSU-974-FAcorr cells used as control and °°, °°°° represent a *p* < 0.01 and *p* < 0.0001 between HU-treated and untreated cells.

*FANCA* gene mutation determines an increment in OHSU-S91 growth compared to OHSU-FAcorr (Figure 8B), although the cell morphology appears similar in both samples (Figure 8A). Moreover, OHSU-S91 displays an increased accumulation of double-strand DNA damages, as indicated by phosphorylated-H2AX (p-H2AX) expression (Figure 8C), both in the absence or presence of hydroxyurea, a molecule favouring DNA damages.

## 4. Discussion

The data reported herein show that *FANCA* mutation causes drastic metabolic changes in an HNSCC cellular model. Specifically, OHSU-S91 cells display the same defect in the electron transfer between respiratory complexes I and III observed in FA lymphoblasts and fibroblasts [24,25], which causes a decrement in the OxPhos efficiency, as shown by the P/O ratio in the presence of pyruvate and malate, and a reduction of disposable chemical energy. 

Although HNSCC cells are already characterized by enhanced glycolysis in normoxic conditions [44], the mitochondrial function impairment due to the *FANCA* mutation further favours the switch to anaerobic metabolism, as demonstrated by the increment of LDH activity and the anaerobic glycolysis yield in OHSU-S91 compared to OHSU-FAcorr cells. *FANCA* mutation also causes a change in affinity for metabolic substrates in OHSU-S91. Our data show that OHSU-FAcorr aerobic metabolism is supported predominantly by glutamine and partly by glucose, confirming the data reported by Yang et al. [45]. By contrast, in OHSU-S91, the mitochondrial activity dysfunction is associated with a drastic reduction in glutamine employment in favour of glucose. Probably, the change in affinity for energy substrates reflects the need for OHSU-S91 to increase the availability of the building blocks needed to support increased cell proliferation. Interestingly, the literature reports that HNSCCs already display mitochondria metabolic dysfunction [45,46] and an increased expression of proteins involved in the glucose metabolism, such as glucose importers and glycolytic enzymes (HK2, PFK, LDH) that have been associated with poor prognosis in HNSCC [47]. HNSCC also displays alterations in lipid metabolism due to the increment of glycoprotein import fatty acid (CD36) and fatty acid synthase, which are associated with advanced disease and adverse prognosis [47]. In addition, HNSCCs with *FANC* mutations show an altered lipid metabolism associated with gangliosides’ overproduction and accumulation, which contribute to the tumour aggressiveness and invasiveness [48]. Therefore, the increased anaerobic metabolism and the altered lipids metabolism with lipid droplets accumulation in FA cells correlated to the mitochondria defect [22] might be responsible for the high proliferation and aggressivity of HNSCC in FA patients. Western blot analyses show that the defect in the OHSU-S91 cells aerobic metabolism does not depend on an altered expression of OxPhos proteins but a defective mitochondrial reticulum dynamic as OHSU-S91 cells over-express DRP1, a protein involved in the fission process compared to OHSU-FAcorr cells. It is known that the aerobic metabolism efficiency depends on the mitochondrial network plasticity maintained by the balance between fusion and fission processes [37,49]. When fission prevails over fusion, the mitochondrial reticulum breaks down, and mitochondria separate, losing part of their energy-producer capacity [50]. In addition, OHSU-S91 cells appear unable to complete the mitophagy processes. In fact, despite a similar expression of PINK1 compared to OHSU-FAcorr, OHSU-S91 cells display a lower expression of PARKIN, an E3 ubiquitin ligase involved in the mitochondrion polyubiquitination. In other words, OHSU-S91 cells tag damaged mitochondria via PINK1 but fail to ubiquitinate them, causing the accumulation of dysfunctional mitochondria. Finally, OHSU-S91 cells also show a defect in triggering autophagy, as the expression of beclin1, a protein inducing the autophagosome formation, appears lower than that of OHSU-FAcorr, despite the downstream effectors being similarly expressed in both cell lines and the LC3 activation being higher in OHSU-S91 compared to the OHSU-FAcorr. In other words, the *FANCA* mutation in HNSCC cell models causes an accumulation of damaged and poorly functioning mitochondria that promote the metabolic switch to anaerobic glycolysis and the production of oxidative stress.

As for *FANCA* lymphoblasts [24], the mutated *FANCA* gene causes a minor activation of endogenous antioxidant defences, specifically of the reduced glutathione (GSH) production pathway. This alteration could favour the HNSCC development since GSH is fundamental for the glutathione S-transferase, an enzymatic family involved in the phase II carcinogen detoxification [51]. On the other hand, it was demonstrated that mutations in glutathione-S-transferase family genes are among those responsible for the head and neck cancer onset [52].

The decrement of endogenous antioxidant defences and the electron transport breakdown causes MDA accumulation in OHSU-S91. Interestingly, HNSCC cancer stem cells counteract elevated aldehyde levels by the over-expression of aldehyde dehydrogenase 1 (ALDH1), which correlates with HNSCC self-renewal and metastatic capacity [53,54]. Since aldehyde accumulation causes a genotoxicity increment in FA cells, HNSCC cells carrying FA mutation can accumulate more DNA damage [55,56], which could favour tumour invasiveness and aggressiveness. This hypothesis is confirmed by the double-strand DNA breaks accumulation in OHSU-S91 compared to OHSU-FAcorr. This data is not surprising considering the pivotal role of FA genes in the DNA repair mechanisms [57].

## 5. Conclusions

These metabolic alterations suggest that the presence of *FANCA* mutation exacerbates the anaerobic metabolism and the relative Warburg effect, which already characterized the HNSCC metabolism [44], inducing cell growth acceleration, as demonstrated by our data. On the other hand, it is known that high proliferative and aggressive cancer cells are characterized by anaerobic glycolysis as energy metabolism despite normoxic environmental conditions to favour the uptake and incorporation of nutrients as building blocks necessary to promote cell proliferation [58]. In addition, uncoupled mitochondria combined with the antioxidant defences decrement determine a slight increase in oxidative stress, representing another factor promoting cell proliferation [59]. In other words, the characterization of metabolic features in HNSCC carrying FA genes mutations could explain the strict correlation between HNSCC and FA gene, helping to identify new therapeutic targets for both diseases.

## Data Availability

The analysed data supporting the conclusions of this article are included within this article and its additional files.

## Supplementary Materials

The following supporting information can be downloaded at: https://www.mdpi.com/article/10.3390/cells11152353/s1, Figure S1: Succinate-induced Complex III assay. Mitochondrial respiratory complex III activity stimulated by the succinate addition. Data are reported as mean ± SD, and graph is representative of at least 3 independent experiments. Statistical significance was tested opportunely with the un-paired *t*-test and no significant differences are observed between OHSU-974-S91 cells and the OHSU-974-FAcorr cells; Figure S2: Whole WB signals.
