# Supplementary material for: Mutated FANCA Gene Role in the Modulation of Energy Metabolism and Mitochondrial Dynamics in Head and Neck Squamous Cell Carcinoma
Source: Cells. 2022 Jul 30;11(15):2353. doi: 10.3390/cells11152353 (PMC9425438; doi:10.3390/cells11152353)
Supplement: Supplementary file 1 [file cells-11-02353-s001.zip › Supplementary Materials_Bertola et al 11.08..pdf]

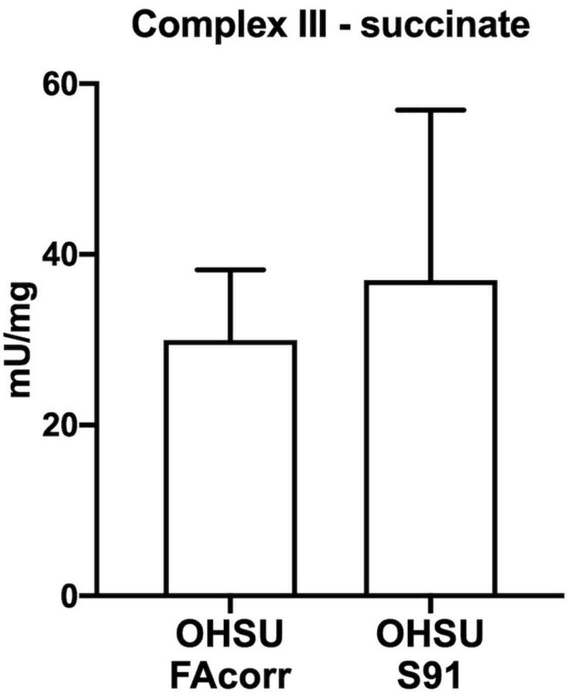

Supplementary Figure S1: Succinate-induced Complex III assay. Mitochondrial respiratory com- plex III activity stimulated by the succinate addition. Data are reported as mean ± SD, and each graph is representative of at least 3 independent experiments. Statistical significance was tested opportunely with the unpaired t-test and no significant differences are observed between OHSU- 974-S91 cells and the OHSU-974-FAcorr cells.

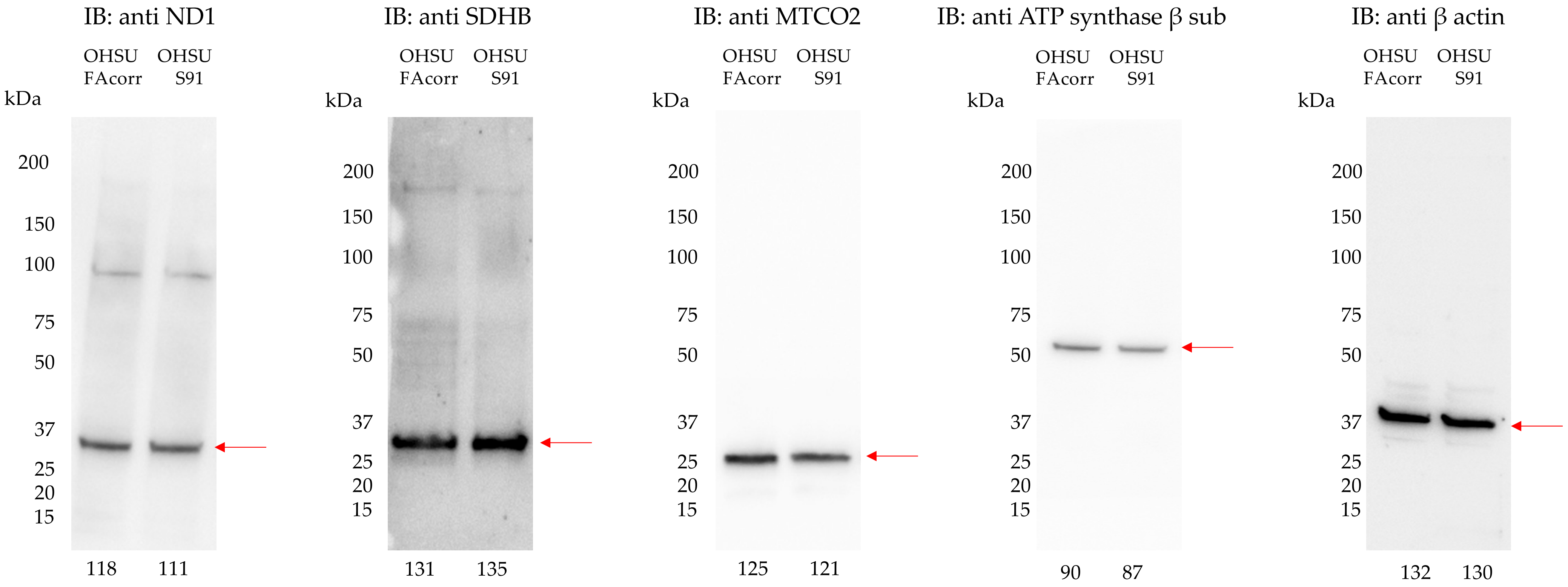

Supplementary Figure S2: Whole Western Blot signals. The figure reports the whole western blot signals corresponding to the cropped images inserted in the main text figures. Each page shows in the top right-hand corner the number of the figure in the main text to which it refers.

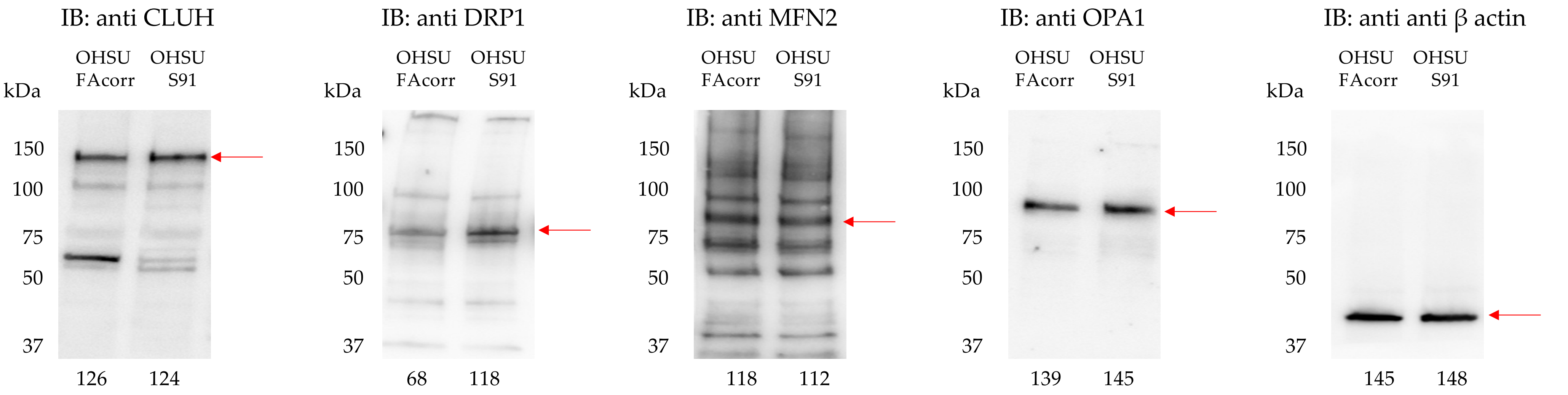

Supplementary Figure S2: Whole Western Blot signals. The figure reports the whole western blot signals corresponding to the cropped images inserted in the main text figures. Each page shows in the top right-hand corner the number of the figure in the main text to which it refers.

WB_Fig6

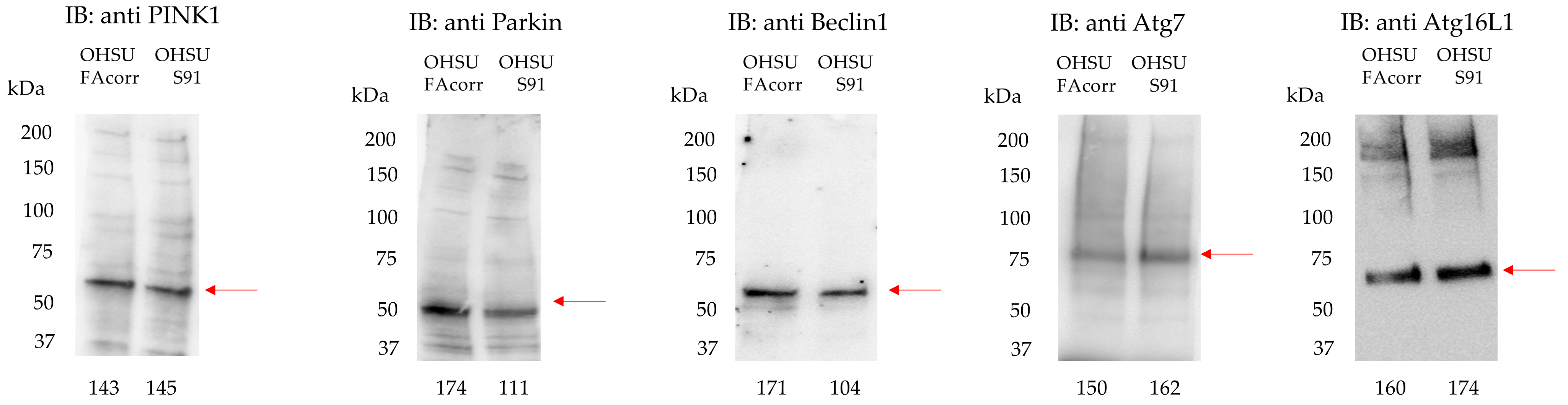

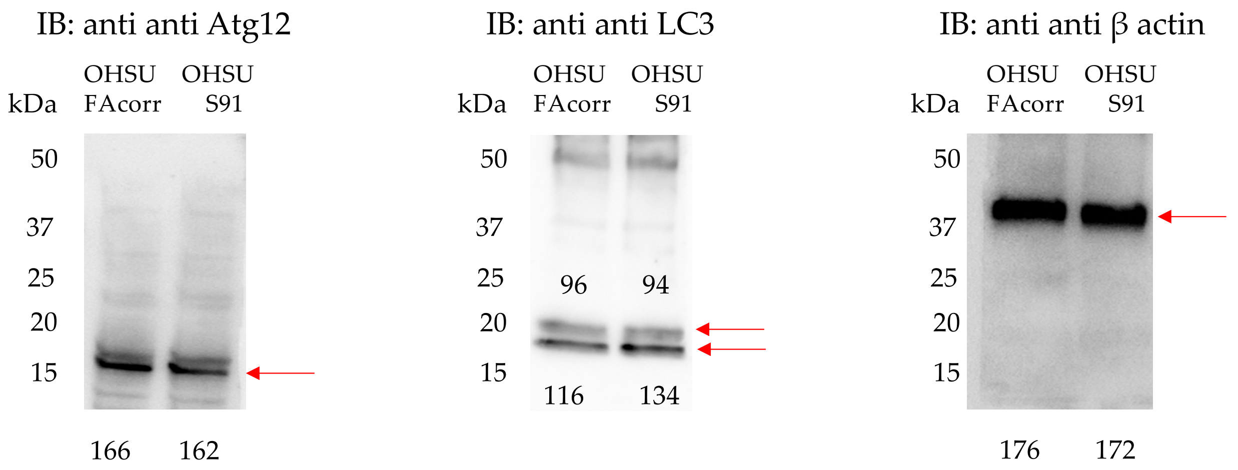

Supplementary Figure S2: Whole Western Blot signals. The figure reports the whole western blot signals corresponding to the cropped images inserted in the main text figures. Each page shows in the top right-hand corner the number of the figure in the main text to which it refers.

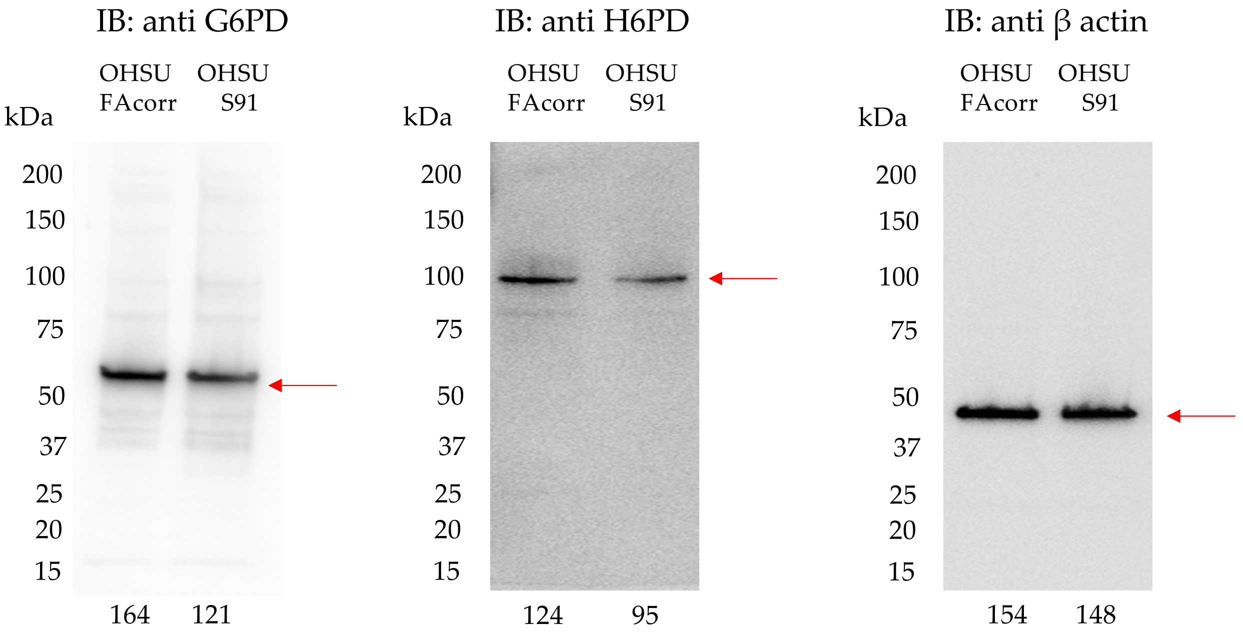

Supplementary Figure S2: Whole Western Blot signals. The figure reports the whole western blot signals corresponding to the cropped images inserted in the main text figures. Each page shows in the top right-hand corner the number of the figure in the main text to which it refers.

WB_Fig8

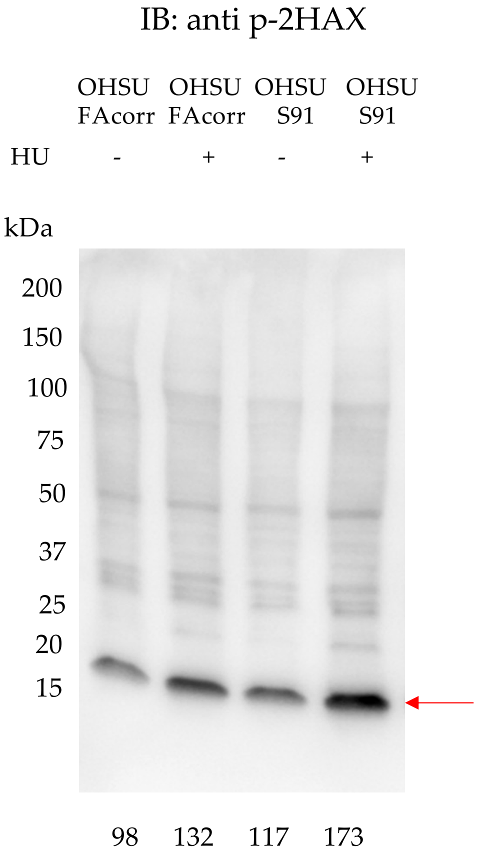

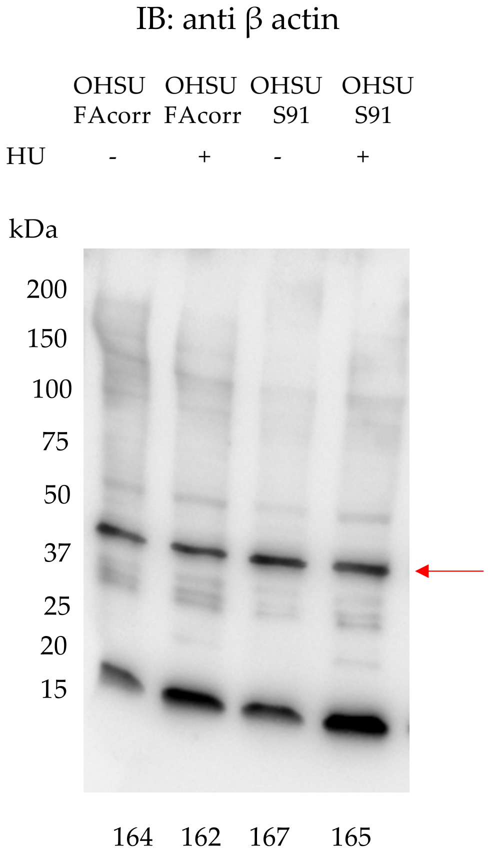

Supplementary Figure S2: Whole Western Blot signals. The figure reports the whole western blot signals corresponding to the cropped images inserted in the main text figures. Each page shows in the top right-hand corner the number of the figure in the main text to which it refers.
